# Comparing screening based on the NHS Health Check and Polypill Prevention Programmes in the primary prevention of heart attacks and strokes

**DOI:** 10.1177/09691413241235488

**Published:** 2024-03-15

**Authors:** Nicholas J Wald, Aroon D Hingorani, Stephen H Vale, Jonathan P Bestwick, Joan Morris

**Affiliations:** 1 Institute of Health Informatics, University College London, London, UK; 2 Population Health Research Institute, 4915 St Georges University of London, London, UK; 3 Institute of Cardiovascular Science, University College London, London, UK; 4 Logical Medical Systems, London, UK; 5 Centre for Preventive Neurology, Wolfson Institute of Population Health, 4617 Queen Mary University of London, London, UK

**Keywords:** Screening, NHS Health Check, polypill, heart attacks, strokes

## Abstract

**Objective:**

To compare the NHS Health Check Programme with the Polypill Prevention Programme in the primary prevention of heart attacks and strokes.

**Design:**

Use of published data and methodology to produce flow charts of the two programmes to determine screening performance and heart attacks and strokes prevented.

**Setting:**

The UK population.

**Intervention:**

The NHS Health Check Programme using a QRISK score on people aged 40–74 to select those eligible for a statin is compared with the Polypill Prevention Programme in people aged 50 or more to select people for a combination of a statin and three low-dose blood pressure lowering agents. In both programmes, people had no history of heart attack or stroke.

**Main outcome measures:**

In 1000 people, the number of heart attacks and strokes prevented in the two programmes.

**Results:**

In the hypothetical perfect situation with 100% uptake and adherence to the screening protocol, in every 1000 persons, the NHS Health Check would prevent 287 cases of a heart attack or stroke in individuals who would gain on average about 4 years of life without a heart attack or stroke amounting to 1148 years in total, the precise gain depending on the extent of treatment for those with raised blood pressure, and 136 would be prescribed statins with no benefit. The corresponding figures for the Polypill Prevention Programme are 316 individuals who would, on average, gain 8 years of life without a heart attack or stroke, amounting to 2528 years in total, and 260 prescribed the polypill with no benefit. Based on published estimates of uptake and adherence in the NHS Health Check Programme, in practice only 24 cases per 1000 are currently benefitting instead of 287, amounting to 96 years gained without a heart attack or stroke.

**Conclusions:**

The Polypill Prevention Programme is by design simpler with the potential of preventing many more heart attacks and strokes than the NHS Health Check Programme.

## Introduction

Screening in the primary prevention of future cardiovascular disease is currently based on performing periodic health checks among adults, based on clinical history, a limited physical examination including blood pressure measurements and a blood test to measure cholesterol, to derive a person's risk of developing cardiovascular disease. Such screening is conducted in several countries including the UK. The risk assessment was originally based on the results of the US Framingham Study^1^ and later adapted using other data. UK primary care data were used to develop the multi-factor QRISK prediction estimator in the NHS Health Check Programme conducted in England for people who are aged 40 to 74.^2^ In the NHS Health Check Programme, QRISK is used to estimate the risk of a cardiovascular disease event over the next 10 years.

Health Checks, which are the responsibility of local authorities, are conducted every 5 years, with some occurring within and some outside general practices. Persons with a 10-year cardiovascular disease risk of 10% or more are deemed screen positive. All individuals are offered advice on lifestyle with prescribing of any medication subsequently being left to GPs, and Public Health England advising that a statin is prescribed ‘where lifestyle modification has been ineffective or is inappropriate’. If the person's blood pressure is not thought to be raised, blood pressure lowering medication is not offered.

In the year 2000, recognising that age overwhelms all other predictors, a simpler method of screening was proposed in a patent application^3^ using age alone, without testing or physical examination. This was brought to wider attention in the BMJ in 2003^4–6^ in what have become known as the ‘Polypill papers’.^7^

A Polypill Prevention Programme has been offered outside the NHS for over 10 years as a service accessible on www.polypill.com. The Polypill Prevention Programme is directly linked to risk reduction where people aged 50 and older, without contraindications, are offered a combined formulation of medicines: a polypill. This consists of a cholesterol-lowering statin and three low-dose blood pressure lowering medicines to lower both risk factors together *regardless of their starting values*. Randomised trials have demonstrated the value of the polypill versus usual treatment with few side effects.^8–12^

The contrasting approaches of the NHS Health Check Programme and the Polypill Prevention Programme raise questions about the future of public health policy on the primary prevention of cardiovascular disease. First, assuming 100% uptake and adherence to both preventive programmes, how do they compare? Second, how does the NHS Health Check Programme perform in practice, where uptake and adherence are unlikely to be 100%, and how is this likely to compare with the Polypill Prevention Programme? Third, how should the benefits of the two approaches be quantified and compared? We here answer these questions using evidence published by others and building on our previous work.^4,13–16^

In this study, we compare NHS Health Check screening with age-alone screening using a flow chart analysis applied to 1000 people in the population, an analysis not previously done, but the results apply to other multiple risk factor-based screening programmes for the primary prevention of heart attacks and strokes, similar to the NHS Health Check, that are being conducted in many parts of the world.

## Methods and results

**Figure 1.**
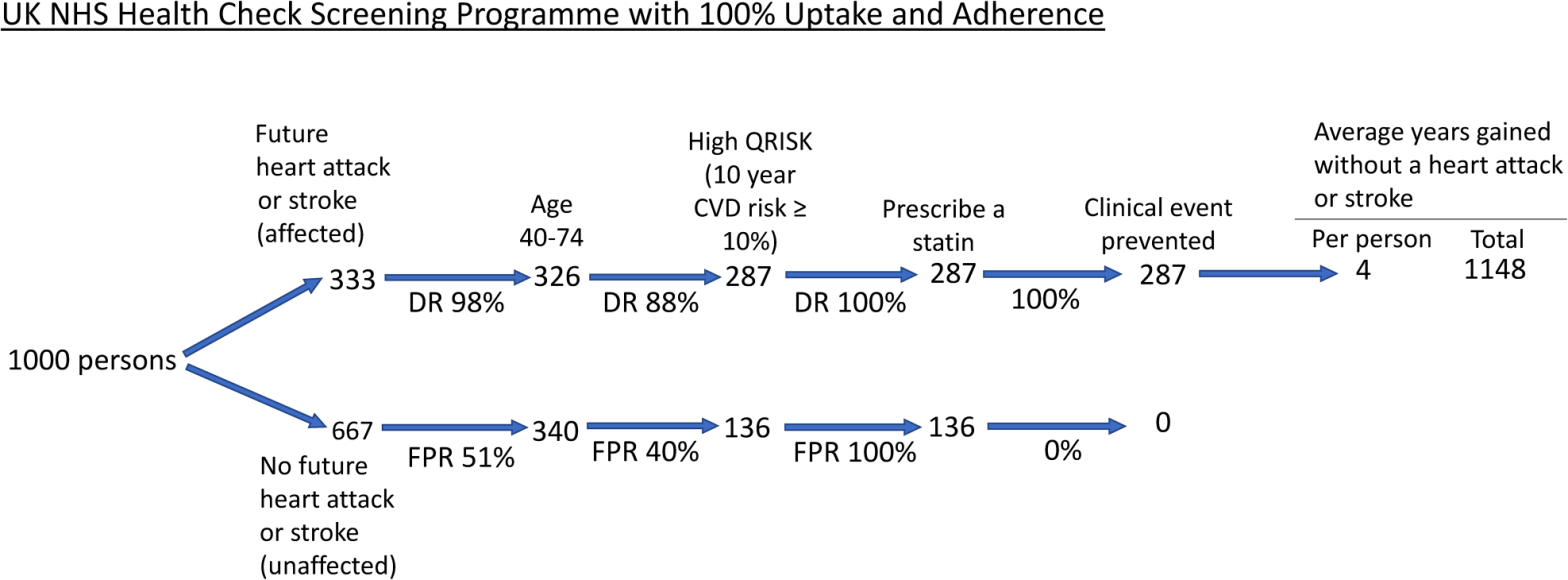
Flow diagram of the NHS Health Check Programme among 1000 people in the population with 100% uptake and adherence. ‘Clinical event prevented’ includes those delayed. DR: detection rate; FPR: false positive rate: CVD: cardiovascular disease.

**Figure 2.**
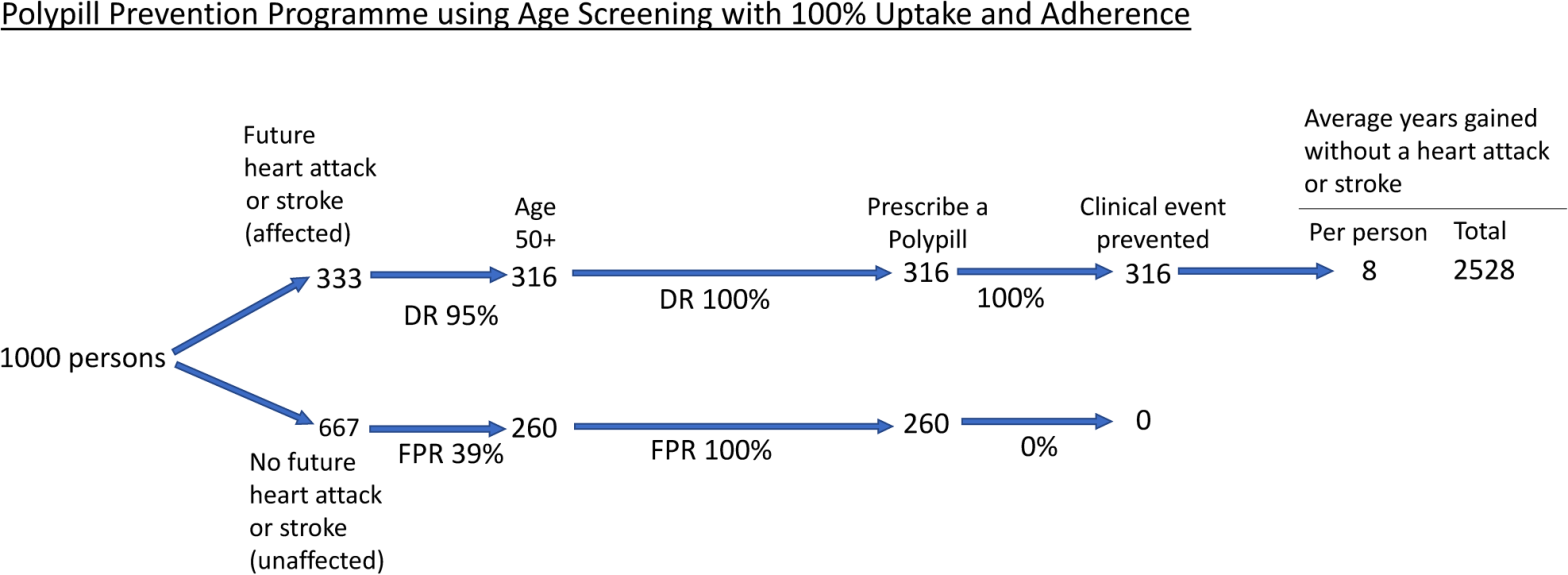
Flow diagram of the Polypill Prevention Programme among 1000 people in the population with 100% uptake and adherence. ‘Clinical event prevented’ includes those delayed. DR: detection rate; FPR: false positive rate.

**Figure 3.**
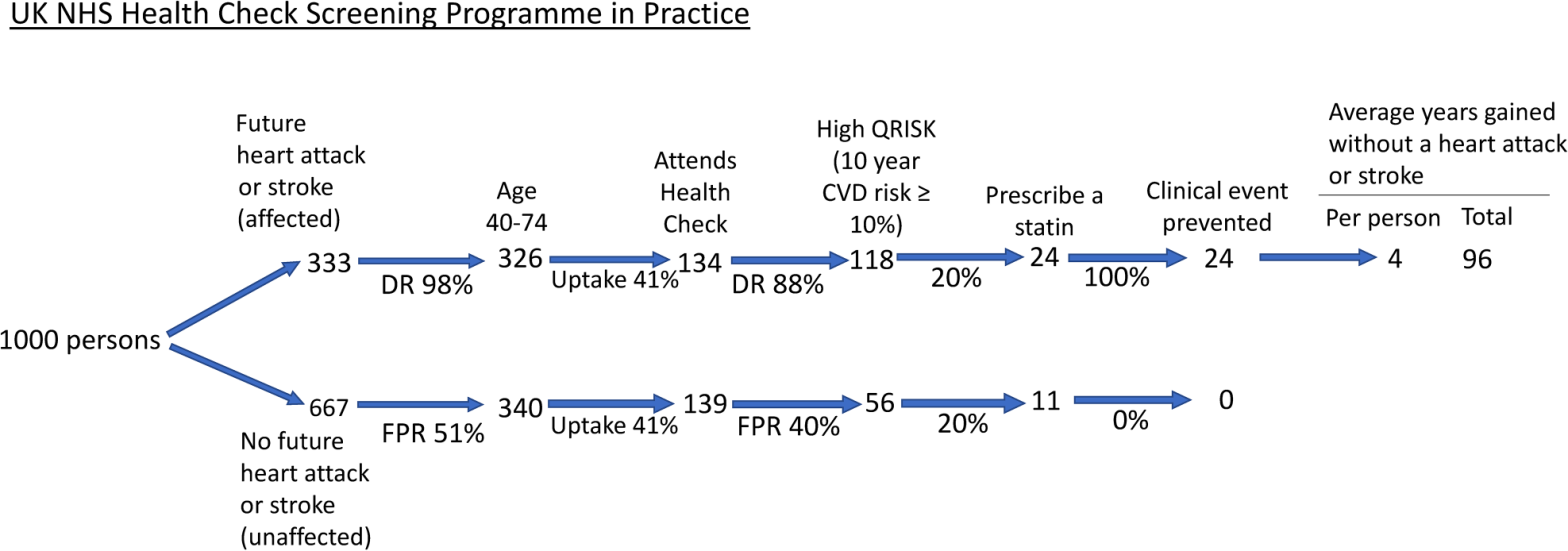
Flow diagram of the NHS Health Check Programme among 1000 people in the population taking account of the observed uptake and statin use. ‘Clinical event prevented’ includes those delayed. DR: detection rate; FPR: false positive rate: CVD: cardiovascular disease.

Published results were used to construct three flow diagrams to show the screening performance and preventive effect in 1000 persons in the population regardless of age, divided into those that have a future fatal or non-fatal heart attack or stroke over their lifetime (affected) and those that will not have either event (unaffected). One flow diagram is for the UK NHS Health Check Programme, and one is for the Polypill Prevention Programme (Figures 1 and 2, respectively). In both flow diagrams, it is taken that there is complete adherence to the respective protocols. The third flow diagram (Figure 3) is for the NHS Health Check, constructed based on results on uptake and statin use from a published audit of the programme.

### NHS Health Check Programme

About 1 in 3 people in the population (333 per 1000) will be affected by a heart attack or stroke over their lifetime and the remainder (667 per 1000) will be unaffected.^14^ The first step is identifying people aged 40–74 years as shown in Figure 1. Standard life-table analysis shows that about 98% of affected individuals (326 of 333) and about 51% of unaffected individuals (340 of 667) will be aged 40 or older^17^; the actual estimates will be only a little less with an upper limit of 74 years as specified in the NHS Health Check Programme (eligible people aged 40–74) because by age 74 almost everyone will be screen positive. The second step in the screening process is identifying those with a positive QRISK score, i.e. a 10-year cardiovascular disease risk greater than or equal to 10%. With 5-yearly assessments, using a 10% 10-year cardiovascular disease risk cut-off, the detection rate is 88% (proportion of individuals with a first cardiovascular disease event in the next 10 years with positive screening results) and the false positive rate is 40% (proportion of individuals who do not have a first cardiovascular disease event in the next 10 years with positive screening results). This yields 287 true positives (88% of 326) and 136 false positives (40% of 340). These estimates are based on the Framingham risk score^13^ which has similar performance to QRISK.^18^ The third, and final, step is prescribing a statin to QRISK positive individuals.

Figure 1 shows that in the NHS Health Check Programme all 287 true positives receive a statin and will, as a result, have a heart attack or stroke entirely prevented or delayed, since medication with a statin will have a preventive effect in all who take it, albeit with a variable preventive period adopting the holistic model described in Wald and Morris.^14^ The holistic model shows that, on average, a gain of 4 years of life without a heart attack or stroke would be achieved with a statin (see Appendix) amounting to 1148 person years gained. These estimates may be somewhat greater depending on the use of blood pressure lowering medications being prescribed in the NHS Health Check Programme if the blood pressure was considered to be high.

Figure 2 shows, in the same way as Figure 1, the results of the Polypill Prevention Programme, which uses age alone in selecting people for preventive medication. Among persons in the affected group shown in the Figure, standard life-table analysis^14,17^ indicates that 95% of first heart attacks and stroke will be found in those aged 50 and over (not much less than the 98% found in those aged 40 and over), yielding 316 true positives. In the unaffected group, life-table analysis indicates that 39% of all people who do not have a first heart attack or stroke will be found among those aged 50 and over, yielding 260 false positives. All 576 individuals (true and false positives) are prescribed a polypill, including a statin and three low-dose blood pressure medicines, with 316 clinical events prevented or delayed, gaining, on average, 8 years of life without a heart attack or stroke^14^ amounting to 2528 person years gained, more than double the number in the NHS Health Check Programme.

### Implementation of the NHS Health Check Programme

NHS Health Checks started in England in 2009. Figure 3 shows a flow diagram taking account of an audited 41% uptake^19^ and a 20% use of statins in QRISK positive individuals.^16^ A subsequent 2021 audit yielded lower uptake and adherence estimates.^15^ Using the estimates of 41% and 20%, respectively, out of 1000 people, 24 cases would be prevented (Figure 3) compared to 287 with full uptake and everyone with a positive QRISK receiving a statin (Figure 1), i.e. 92% fewer. The person years of life gained without a heart attack or stroke would be 96.

### Comparison of the programmes

Table 1 shows the differences between the two screening programmes with 100% uptake and protocol adherence. In every 1000 people, the Polypill Programme prevents 29 more heart attacks and strokes (316 minus 287) than the NHS Health Check Programme with 124 more false positives (260 minus 136) of negligible clinical significance given the safety of the preventive medication and, importantly, *no clinic visits or blood tests*. But in practice, uptake and protocol adherence of the Health Check Programme is far from 100%. Despite the uptake in a Polypill Prevention Programme not being known, using the audit results of the NHS Health Check Programme indicates that uptake in a Polypill Programme would have to be only 8% (24/316, from Figures 2 and 3) to achieve a better outcome in terms of clinical events prevented.

**Table 1.** Comparison of NHS Health Check Programme and Polypill Programme among 1000 people in the population with 100% uptake and adherence.

		NHS Health Check Programme	Polypill Prevention Programme
1.	(i) In every 1000 people: number of heart attacks or strokes prevented	287	316
	(ii) Receiving medication, who would or would not have had a heart attack or stroke	136	260
	(iii) Number of clinic visits	7000^a^	0
2.	No. of screening steps	3 (age, QRISK, prescribe a statin)	1 (age only)
3.	Preventive medication	Statin with or without blood pressure lowering medication	Statin and low-dose blood pressure medication (Polypill)
	Years of life gained without a heart attack or stroke		
	(i) Years per person	4^b^	8
	(ii) Total	1148	2528
4.	Uptake and adherence	Very low	Unknown

^a^ 1000 × 7 for seven 5-yearly ‘Health Checks’.

^b^ With statin only.

## Discussion

This paper uses the NHS Health Check Programme as an example of a cardiovascular risk algorithm that has been shown to have a similar screening performance to others, including the Framingham algorithm.^18^ Our results, therefore, apply to other widely adopted risk algorithms. Consequently, the conclusions of our paper extend beyond UK practice.

The flow charts in Figures 1 and 2 show that with complete uptake and adherence in both programmes, the Polypill Prevention Programme prevents more heart attacks and strokes than the NHS Health Check Programme without requiring clinic visits or blood tests. Figure 3 shows that with the reported uptake of the NHS Health Check Programme and the number of people prescribed a statin the Programme has a very limited preventive effect. The analysis involved in the flow charts has the advantage that it is simple and transparent, based as it is on published results and methodology. Even in the hypothetical perfect situation, the Polypill Programme is more effective and likely to be much more effective in practice. Consideration needs to be given to replace the NHS Health Check Programme with an audited population-based Polypill Prevention Programme. In making such a switch in policy, several issues in regard to each programme need to be considered, as covered below.

### NHS Health Check approach

The NHS Health Check Programme is a three-step screening programme. First, people are selected based on age (40–74). Then tests are performed to determine a person's 10-year risk of a cardiovascular disease event. This decreases the false positive rate, but also decreases the detection rate. Third, a clinical decision is made on whether a statin is prescribed, possibly only after attempting to reduce risk through diet and lifestyle, and separately, based on blood pressure measurements, a clinical decision may be made on whether a blood pressure lowering medication is prescribed. However, where a preventive intervention is safe and affordable, as is the case with statins and blood pressure lowering medications,^5,6,20^ the balance should be in favour of a simple approach that increases detection and disease prevention, employing a policy that directly offers access to preventive medication.

Aside from the low uptake and adherence to the NHS Health Check Programme, there is another weakness. The aim of preventive medication should be to reduce the risk of cardiovascular disease as much as possible, which is achieved by lowering blood pressure *and* LDL (low-density lipoprotein) cholesterol together, not just one or the other if either are thought to be abnormally high, and to do so *regardless of the starting level* because there is no practical blood pressure threshold below which there is no further reduction in risk.^21–24^

A 10-year risk of a heart attack or stroke equal to or greater than a given percentage (say ≥ 10%) is too limited for assessing both risk and benefit in chronic disease prevention, where the risk of disease is lifelong and requires lifelong preventive intervention. A further consideration relates to missed preventable cases in the NHS Health Check Programme. If a person has a positive QRISK assessment (i.e. ≥10% 10-year risk), preventive medication is offered. Once started it will, presumably, be taken for the rest of that person's life. From about age 74 almost everyone will be QRISK screen positive. The effect of the NHS Health Check Programme will be to miss the opportunity to prevent some heart attacks and strokes at younger ages that could have been prevented with an age-alone policy. For example, a 60-year-old could have a negative assessment, but on an age-alone policy would be positive and eligible for preventive medication.

There is minimal benefit in starting preventive medication below age 50 as is the case in the NHS Health Check Programme. Selecting age 50 as the age a polypill is offered is a policy judgement that may vary from country to country depending on the age distribution of heart attacks and strokes, cost and affordability. This is because there are very few people below age 50 who have a positive QRISK (reflecting the fact that age is the most important determinant of risk), very few events occur between age 40 and 50, and the full effect on risk reduction from blood pressure and LDL cholesterol reduction is achieved after only about 3 years.^5^

In 2014, the NHS Health Check Programme lowered the 10-year cardiovascular risk threshold from 20% to 10%, but this led to little change in practice; the average calculated risk of all people who were started on statins was about 21% before the guideline was changed and about 20% after.^16^ This calls into question what the programme achieves and indicates that the policy was not followed.

In a survey of 1.4 million people registered with 248 UK general practices, 73% of individuals initiated on a statin did not have a QRISK score recorded at any time (37,215/50,940).^25^ On this basis alone, it can be concluded that a multi-factor screening ‘tool’, such as QRISK, plays only a minor role in influencing the decision to start people on a statin.

Importantly the results in Figure 3 show that the Health Check Programme has a very small effect, with the unavoidable conclusion that it should be replaced by a more effective programme.

### Polypill Prevention approach

Apart from already having had a cardiovascular event, the overriding influence on a person's future risk of a cardiovascular event is that person's age. From youth to old age, the risk of cardiovascular events doubles every 7–8 years.^26^ Other risk factors, like blood pressure and LDL cholesterol, although causal and reversible, are poor predictors of disease.^27^ It follows that in the primary prevention of cardiovascular disease, age alone can be used in a once-only screening enquiry. There is only a marginal improvement in screening performance over age alone from adding causal risk factors.^13^ A person's age is already available from that person's medical records. Age alone is the screening test that determines eligibility for preventive medication that can then be offered in the absence of specific medical contraindications. Indeed, while selection on age is a critical first screening test for almost all screening programmes, it is not recognised as such, but its importance is shown in the Figures as a critical step even in the NHS Health Check screening pathway.

Appropriately formulated with a statin and low-dose blood pressure medications, a polypill has a low incidence of side effects. In a crossover trial, there were no withdrawals due to side effects.^20^ Comprehensive analysis on statins indicated side effects were rare.^28^ The use of blood pressure medication at low doses in randomised trials showed a very low rate of side effects^6^ and a reduction of one third in headache^29^ which was supported by a survey of Polypill participants in 2020 (https://www.polypill.com/Home/Headaches). Importantly, side effects are reversible on stopping treatment and vastly outweighed by the benefit. In the UK Polypill Prevention Programme less than 1% of the participants discontinued on account of side effects, all of which were minor, and not necessarily related to taking the Polypill. The safety profile and low cost of the preventive medication justifies accepting a simpler and more effective policy, even though it results in more people who would not have a heart attack or stroke receiving the medication.

High cost and potential harm are reasons to limit the offer of a medical intervention to screen-positive individuals selected based on precisely determined high-risk estimation. But this is not the case if prevention is inexpensive and safe as it is with a polypill, justifying the conclusion that screening can be based on age alone, with a starting age just before heart attacks and stroke become a significant disease burden in the population. In such a situation, prevention is better than prediction. Costs would be largely limited to producing the polypill and making it accessible to the public, not adopting an expensive complex protocol with repeated measurements every 5 years to estimate the risk of disease; the NHS Health Check has been shown to take typically 20–30 min for each assessment^30^ and a report in 2015 estimated that the annual cost was £450million.^31^

A well-managed Polypill Prevention Programme conducted at scale would be an effective, low cost, safe medical intervention that would, to advantage, replace the NHS Health Check Programme. As well as the health benefits,^14^ the programme would be cost effective.^32^ Uptake and adherence are important. Figures 1 and 2 describe a hypothetical perfect situation with 100% uptake and medication adherence to each of the two programmes recognising that in practice this will be less. Figure 3 shows the practical effect of the NHS Health Check Programme. Comparable estimates for uptake and adherence are not available for a Polypill Prevention Programme because the programme in the UK is based on individuals being aware of the service and choosing to become a participant and, therefore, not representative of what would happen in the general population. However, the effectiveness of a Polypill Programme is likely to be significantly greater than the NHS Health Check Programme for three reasons. Firstly, the uptake and adherence in a Polypill Programme would only have to be as low as 8% to be better than the NHS Health Check Programme. Secondly, a Polypill Programme requires little or no inconvenience to people offered such a service through their family doctor or community pharmacy on reaching the age of 50. Thirdly, it has been shown, in several studies that are summarised in a meta-analysis of randomised trials, that polypills improve adherence to treatment over usual treatment.^33^ In any event, monitoring uptake and adherence would be needed. We are aware of no other public health measure that would currently have as large an impact on the primary prevention of disease as the Polypill Prevention Programme.

## Conclusion

The NHS Health Check Programme is less effective than the Polypill Prevention Programme in the primary prevention of heart attacks and strokes. Replacing the NHS Health Check Programme, and similar programmes around the world, with Polypill Prevention Programmes would secure significant health benefits to individuals and populations.

## Appendix

### Source of estimates used in the figures

**Figure 1.** About 333 people of the initial 1000 will be affected with a heart attack or stroke over their lifetime^14^ and 667 will be unaffected. Approximately 98% of affected individuals (326) and 51% of unaffected individuals (340) will be aged 40 or more years^17^ and so eligible for the NHS Health Check. Of these, approximately 88% of affected individuals (287) and 40% of unaffected individuals (136) aged 40 to 74 will have a cardiovascular disease risk greater than 10%.^13^ All these people will be prescribed a statin and so 287 will have a clinical event prevented. On average, people prescribed a statin will gain 4 years of life without a heart attack or stroke^14^ and hence, 287 × 4 such years of life gained in a population of 1000 people. The 4 years of life gained was derived as described in Wald and Morris,^14^ using age-specific relative risk reductions for a conservative LDL cholesterol reduction of 1.54 mmol/litre for heart attacks (e.g. about 55% at age 60) and a 15% reduction for stroke.^5^

**Figure 2.** As Figure 1 but with approximately 95% of affected individuals (316) and 39% of unaffected individuals (260) aged 50 or more^17^ and so eligible to join the Programme. These people will be prescribed a polypill consisting of a statin and three low-dose blood pressure lowering medicines thus preventing 316 clinical events. On average, people prescribed the polypill will gain 8 years of life without a heart attack or stroke^14^ and hence, 316 × 8 such years of life gained in a population of 1000 people.

**Figure 3.** As Figure 1 but with a 41% uptake^18^ of the NHS Health Check and 20% of QRISK positive individuals receiving a statin,^16^ thus preventing 24 clinical events. On average, people prescribed a statin will gain 4 years of life without a heart attack or stroke,^14^ and hence, 96 (24 × 4) such years of life gained in a population of 1000 people.

### Quantifying the health benefits of the two screening programmes

The Figures show the health benefits of using the previously described holistic model instead of the less appropriate reductionist model,^14^ such as the 10-year risk of a heart attack or stroke because the 10-year risk period is arbitrary, while the risk of disease is lifelong requiring lifelong preventive medication and ignores the preventive effect of delaying the occurrence of a heart attack or stroke. In the reductionist model, the relative risk reduction is used to identify two separate groups: one group consisting of the number of people who have a clinical event that the intervention is designed to prevent and another group that does not; one group experiences all the benefit and the other experiences no benefit at all. In the holistic model everyone who would have had a heart attack or stroke in the absence of treatment benefits because blood pressure and/or LDL cholesterol-lowering treatment will always delay such an event, albeit to a varying extent; it recognises that a clinical event delayed is a preventive benefit. The reductionist model counts only people who do not have a heart attack or stroke as benefitting, without taking account of time gained. Two metrics summarise the quantitative benefit using the holistic model. The first is the lifetime probability of a person benefitting from the intervention. With preventive medication starting at age 50, 1 in 3 people will benefit, which is approximately the proportion of people in the population who will have a heart attack or stroke because heart attacks and strokes are rare under age 50. The second is, among those who benefit, the average gain in life without a heart attack or stroke, which, for an appropriately formulated polypill, is 8 years.^14^ Using a statin alone, about 4 years of life are gained without a heart attack or stroke.
